# Representation to the accident and emergency department within 1-year of a fractured neck of femur

**DOI:** 10.1186/1749-799X-6-63

**Published:** 2011-12-21

**Authors:** David J Bryson, Scott Knapp, Rory G Middleton, Murtuza Faizi, Hardik Bhansali, Chika E Uzoigwe

**Affiliations:** 1 Department of Trauma and Orthopaedics, Leicester Royal Infirmary, Infirmary Road, Leicester LE1 5WW, UK; 2 Department of Trauma and Orthopaedics, Kettering General Hospital, Rothwell Road, Kettering, Northamptonshire NN16 8UZ, UK

**Keywords:** Fractured neck of femur, morbidity, mortality, falls, fragility fracture

## Abstract

**Background:**

The fractured neck of femur (NOF) is a leading cause of morbidity and mortality. The mortality attendant upon such fractures is 10% at 1 month and 30% at one year with a cost to the NHS of £1.4 billion annually. This retrospective study sought to examine rates and prevailing trends in representation to A&E in the year following a NOF fracture in an attempt to identify the leading causes behind the morbidity and mortality associated with this fracture.

**Methods:**

1108 patients who suffered a fractured NOF between 1 January 2002 and 31 December 2007 were identified from a University Hospital A&E database. This database was then used to identify those patients who represented within 1-year following the initial fracture. The presenting complaint, provisional diagnosis and the outcome of this presentation were identified at this time.

**Results:**

234 patients (21%) returned to A&E on 368 occasions in the year following a hip fracture. 77% (284/368) of these presentations necessitated admission. Falls, infection and fracture were the leading causes of representation. Falls accounted for 20% (57/284) of admissions; 20.7% of patients were admitted because of a fracture, while 56.6% of admissions were for medical ailments of which infection was the chief precipitant (28% (45/161)).

**Discussion:**

The causes for representation are varied and multifactorial. The results of this study suggest that some of those events or ailments necessitating readmission may be obviated and potentially reduced by interventions that can be instituted during the primary admission and continued following discharge.

## Introduction

The fractured neck of femur is a leading cause of morbidity and mortality. Hip fracture places considerable physical and physiological strain on the patient and upon the resources of the NHS. In 1990 there were an estimated 1.3 million hip fractures worldwide [1]. With a population increasingly surviving into later decades of life this figure is set to grow by more than 480% to reach 6.3 million by 2050 [2]. Estimates place the incidence of neck of femur fractures at 70,000 - 86,000 per year in the UK [1,3] with an average cost to the NHS of £1.4 billion annually [4]. The mortality attendant upon such fractures is 10% at 1-month and 30% at one year [3]. With the UK incidence of hip fractures anticipated to breach 100,000 cases within the next decade [5] the demands placed upon a service already operating at, or close to, full capacity are only set to increase.

As clinicians and surgical practitioners we are routinely called upon to discuss the pros and cons, the intended benefits and risks of particular surgical procedures with patients and their families. While the epidemiology and pathology of venous thromboembolism, wound infection or neurovascular damage are easy to describe and account for, the morbidity and mortality associated with hip fracture is not so easy to explain. Despite surgical fixation nearly one third of patients will suffer declining health and die within one year. While the procedure may be curative and the fracture fixed and stabilised, a successful outcome is not guaranteed. When relatives enquire about prognosis and the reason for this 30% mortality an answer is not always readily available. The reason for this is unclear--what accounts for the morbidity and resultant mortality?

In an attempt to understand and identify the leading causes and factors implicated in this morbidity we undertook a retrospective review examining the prevalence and causes for representation to acute medical services in the year following a hip fracture.

## Methods

We used an A&E coding database to identify patients who had presented to a University Hospital emergency department with a fractured neck of femur between 1 January 2002 and 31 December 2007. This is a computerised database on which all A&E data, including time and date of presentation, investigations, diagnosis, and departure destination (from A&E), are logged by the A&E medical team. This database was then used to identify those patients from this hip fracture cohort who represented to this same A&E department within 1-year of the fracture. Data on the presenting complaint, provisional diagnosis, and outcome (admission/discharge home/referral to other services) was obtained at this time.

## Results

**Figure 1:**
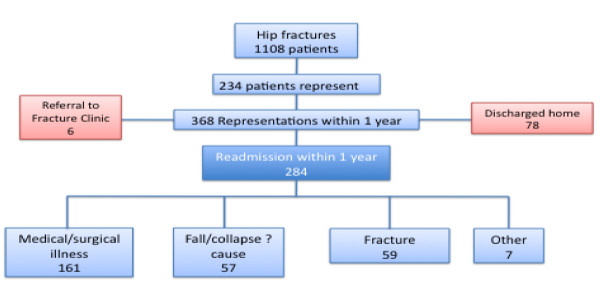
**Outcome of patients representing within 1-year and causes of readmission**.

1108 patients who suffered a fractured neck of femur between 1 January 2002 and 31 December 2007 were identified from the A&E database. 234 of these patients represented to A&E on a total of 368 occasions within 1-year of their original presentation with a hip fracture; 77% (284/368) of the representations necessitated acute admission (Figure 1). Falls, including a collapse of uncertain aetiology, accounted for 20% (57/284) of admissions; 59 patients (20.7%) were admitted with a fracture, of which 23 were to the contralateral hip. Head injury necessitated acute admission on 7 occasions (7 patients; 2.5% of admissions). Medical illness was the leading cause of admission (56.6% (161/284)) of which infection was the chief precipitant (28% (45/161)). Cardiovascular pathology was implicated in 5.3% (15/284) of admissions with five patients (1.8%) presenting with a myocardial infarction or acute coronary syndrome; cardiac arrhythmia was the underlying cause for 2.8% (8/284) of admissions and symptomatic cardiac failure for 0.70% (2/284). Cerebrovascular events (stroke and TIA) accounted for 5.6% (16/284) of admissions. Other less common causes of medical readmission included acute confusion (2.2.8%; 8/284), social incapacity (1.4%; 4/284), and deliberate self-harm (1.8%; 5/284) (Table 1). 50% of those patients who represented within 1 year attended A&E within 4.5 months of the original injury.

**Table 1:** Causes of readmission

Cause	Number		
**Fall/collapse? cause**	57		

**Fracture**	59		
		Contralateral hip	23
		Wrist	5
		Pubic rami	3
		Humerus	2
		Vertebra	2
		Femus	2
		Other	22

**Head Injury**	7		

**Medical/surgical illness**	161		
		**Cardiovascular**	
		Myocardial infarction/ACS	5
		Cardiac failure	2
		Arrhythmia	8
			
		**Infection**	
		Chest	19
		Urinary tract	9
		Skin	5
		Wound	1
		Septicaemia/sepsis? cause	11
			
		**Neurological/Psychiatric**	
		CVA/TIA	16
		Confusion	7
		Deliberate self-harm	5
			
		**Other**	73
		Gynaelogical disorders, social incapacity...	

## Discussion

The orthopaedic surgeon possesses a tried and trusted armamentarium of options for the fixation of hip fractures. Irrespective of the technique employed the goal of surgery is the same--it is undertaken in the hope that it will afford the patient pain relief and the possibility, or opportunity, to return to pre-injury mobilisation levels. Despite the best efforts of the surgical and multidisciplinary teams a significant proportion--nearly 1/3 of patients--will suffer declining health and die within a year. Studies suggest that men and women who sustain a hip fracture have a 8-fold and 5-fold increase respectively in the relative likelihood of death within the first 3 months when compared with age- and sex-matched controls [6]. For those that do survive, 10% will be unable to return to their previous residence [7] and many more will endure a loss of independence requiring formal or informal care provided by social support services or family and friends. Exactly what contributes to this progressive demise is unclear.

In decades past the quality of service provision may have been implicated; the management of osteoporotic or fragility fractures, of which hip fractures are the most physically and medically challenging, has traditionally been sub-optimal [5]. However, the past decade bore witness to considerable changes in the approach to patients with hip fractures. On the basis of work undertaken by the British Orthopaedic and Geriatric Societies, and with the inception of the National Hip Fracture Database in 2007, there now exists evidence-based guidance on the management of hip fractures with emphasis placed upon the establishment of multidisciplinary care plans and secondary prevention. Patients who suffer a hip fracture should expect to receive care that is compliant with the six standards outlined in the be *Blue Book on the care of patients with fragility fractures*, including access to acute orthogeriatric medical support from the time of admission and multidisciplinary assessment and intervention to prevent future falls [5]

The results of this review revealed that 21% of patients returned to A&E in the year following their hip fracture with 77% of these return visits necessitating acute admission. A fall was directly implicated in readmission on 57 occasions and may have been implicated in a further 66 admissions - 59 patients were admitted with a fracture and 7 patients with a head injury. Exactly how these injures were sustained is not known, but it would not be implausible to suggest that a fall may be linked to some of these readmissions. If this were indeed the case, if a fall was the underlying mechanism, it would mean that just under half of all patients who were re-admitted (43%; 57 known falls, 57 fractures, 7 head injures) required acute care because of a fall. A fall may not have been the offending mechanism in all cases--some patients may have sustained pathological fractures or injures following a road traffic accidents--but given the age group and population who most commonly suffer hip fractures there must be a high index of suspicion.

Studies suggest that one half of individuals over the age of 65 will suffer a fall each year and in over 50% of cases the falls will be recurrent [8]. The risk of falling increases with age and with admission into medical and long-term care institutions [9]. Secondary prevention of falls has emerged as a tenet of the multidisciplinary management of hip fractures. With a multitude of factors implicated, including symptomatic cardiovascular pathology such as carotid sinus hypersensitivity or dysrhythmias, along with age related visiospatial decline and muscular deconditioning, a comprehensive assessment of the underlying cause (continuous ambulatory ECG monitoring, tilt-table testing etc) and implementation of appropriate management and educational strategies are indicated. If the cause can be identified, prevention of further falls and reduction in the associated morbidity is a possibility. The primary admission should therefore be considered an opportunity to identify those at risk patients and implement health care initiatives to minimise this risk and maximise health.

While secondary prevention may target falls risk and osteoporotic fragility fractures, these prophylactic or protective measures cannot address all facets of illness and morbidity. Medical ailments were the leading cause of readmission (57%; 161/284) in this study with an infective precipitant identified in 28% (45/161) of cases. Little can be done to prevent individuals from developing pneumonia or a urinary tract infection, yet it is such illnesses, more than any other cause or complaint, that brought this hip fracture cohort back to A&E. Similarly, cardiovascular and cerbrovascular pathology accounted for 11% of re-admissions. The orthogeriatric team may be able to optimise patients medically in preparation for surgery but this is by no means permanent. The progressive decline in health demonstrated by 30% of patients is likely an irreversible process that has been accelerated by the hip fracture. As Goldacre et al. point out, the mortality may be attributed to continuing fracture sequleae but may also be due to the fact that individuals who fracture their hips are more frail and ill than the general population of the same age [10]. In such patients the hip fracture could be a 'tipping point', an insult for which the body doesn't have the reserves to overcome.

The morbidity associated with hip fractures is variable and multifactorial. The results of this retrospective review reveal that in a cohort of patients returning to A&E within 1-year of the original fracture, one-fifth were admitted because of an event that may have been anticipated and the risk reduced with measures (i.e. referral for falls assessment) instituted at the time of original admission. This study did not look at whether any patients were referred for falls assessment but it demonstrates the importance of complying with those standards outlined in the *Blue Book on the care of patients with fragility fractures*. It illustrates the need to look upon the original admission as a opportunity to identify at risk patients and institute measures to optimise health and prevent re-injury and readmission; we should look upon patients with a hip fracture as a 'captive cohort', a proportion of an aging population on whom we can apply targeted measures--medical, educational and social--in an attempt to improve or maximise health and minimise the progressive decline that affects nearly 1/3 of all hip fracture patients.

We readily accept that this review has a number of limitations. Firstly, it does not take into account the morbidity and mortality of the 69% of hip fracture patients who did not present to the Leicester Royal Infirmary A&E. Many patients will be managed in the community and possibly even admitted to community hospitals rather than acute medical services. Similarly, we did not take into account those who may have left the catchment area and therefore may have presented to other A&E departments. Secondly, this review did not examine the locale from which patients were presenting. Nursing home residents or those in residential homes who receive regular skilled care and assistance may have lower rates of attendance compared with those who return to their own home or are reliant upon social service provision or informal care by friends or family. Evidence of such discrepancy may account for the 1.4% of admissions due to social capacity. Lastly, this review utilised an emergency department database to identify patients who suffered a fractured neck of femur and the proportion of this cohort who represented over the following year. Because of variations in coding this database has not yielded details on every neck of femur fracture and the 1108 patients included in this study are therefore a representative sample of hip fracture patients seen in our institution. Similarly, this database cannot provide exact details about the mechanism of injury and for those patients who returned with a fracture we do not know if this was a fragility fracture sustained following a fall from standing height or the result of poly-trauma.

## Conclusion

The morbidity and mortality associated with hip fractures is well documented. This review sought to examine the prevalence and causes for representation and readmission to acute medical services in the year following a hip fracture in an attempt to identify the leading causes behind this morbidity. The results of this review revealed that the causes of representation and readmission are variable and frequently multifactorial. Falls, fracture and infection were the leading causes of readmission. One-fifth of readmissions, possibly more, may have a modifiable aetiology. This illustrates the importance of identifying at risk patients during their original presentation and instituting guidance--namely referral for falls assessment--outlined by the British Orthopaedic and Geriatric societies. Further work is indicated to examine if those patients who actually undergo falls assessment have a lower prevalence of falls and associated morbidity than those who do not.

## Competing interests

The authors declare that they have no competing interests.

## Authors' contributions

Study Design: CU, RM, MR, DB, SK, HB

Data collection: CU, SK

Data Analysis: DB, CU, RM, HB, SK, MF, DB

Writing of the paper: DB, CU, RM, HB, SK, MF

All authors read and approved the final manuscript

## Author Information

Mr David J Bryson MRCS Core surgical trainee

Dr Scott Knapp MB BS Core medical trainee

Mr Rory G Middleton MRCS Specialist Registrar Trauma and Orthopaedics

Mr Murtuza Faizi MRCS Specialist Registrar Trauma and Orthopaedics

Mr Hardik Bhansali MRCS Specialist Registrar Trauma and Orthopaedics

Mr Chika E Uzoigwe MRCS Specialist Registrar Trauma and Orthopaedics
